# Immune-mediated blood-brain barrier disruption after ischemic stroke: mechanisms and therapeutic targets

**DOI:** 10.3389/fimmu.2025.1716041

**Published:** 2025-12-10

**Authors:** Hanjing Wang, Zhang Yiqiang, Jie Cai, Jiawei Guo

**Affiliations:** 1 Department of Vascular and Endovascular Surgery, The First Affiliated Hospital of Yangtze University, Jingzhou, China; 2 Department of Pharmacology, School of Medicine, Yangtze University, Jingzhou, China; 3 Department of Anesthesiology, Jingzhou Hospital Affiliated to Yangtze University, Jingzhou, China

**Keywords:** ischemic stroke, blood-brain barrier, immune inflammation, neutrophil, pro-inflammatory factor

## Abstract

Ischemic stroke is an acute cerebrovascular disorder characterized by the obstruction of cerebral arteries, leading to focal cerebral ischemia and infarction, ultimately resulting in neurological deficits. Its pathogenesis involves a cascade of immune-inflammatory responses and blood-brain barrier (BBB) disruption. Emerging evidence highlights that immune inflammation is a central driver of post-stroke brain injury. Microglial activation, neutrophil infiltration, and the release of pro-inflammatory cytokines collectively exacerbate BBB breakdown and neuronal death. Concurrently, these immune processes participate in tissue remodeling and repair. Notably, the interplay between immune-mediated inflammation and BBB damage forms a vicious cycle that aggravates neurological outcomes and hampers recovery. This review focuses on the molecular mechanisms of ischemia and hypoxia-induced BBB dysfunction, and the immunological processes involved, aiming to provide insights into multi-target and temporally precise neuroprotective strategies for ischemic stroke.

## 1 Introduction

Ischemic stroke remains one of the foremost causes of long-term disability and mortality globally. Although its incidence exceeds that of hemorrhagic stroke, the associated mortality rate is comparatively lower (1). With increasing global aging and a rising prevalence of hypertension and other vascular risk factors, the incidence of ischemic stroke is steadily increasing, placing a disproportionately high burden on low- and middle-income countries (2). While reperfusion therapies—such as intravenous thrombolysis and endovascular thrombectomy—have significantly improved clinical outcomes, their efficacy is often compromised by secondary complications, particularly BBB disruption, hemorrhagic transformation, and cerebral edema (3). Recent studies have demonstrated that immune-inflammatory responses play a dual role in the pathophysiology of ischemic stroke, functioning as both injurious and reparative agents (4). During the acute phase, activated microglia and infiltrating peripheral immune cells produce a surge of pro-inflammatory mediators that aggravate neurological deficits by compromising BBB integrity, inducing neuronal apoptosis, and impairing synaptic plasticity (5). Conversely, during the resolution phase, a regulated inflammatory response is essential for clearing cellular debris, promoting neurotrophic factor secretion, and initiating angiogenesis and neuroregeneration (6). This paradoxical role of the immune system—simultaneously contributing to injury and facilitating repair—provides a compelling rationale for the development of temporally specific immunomodulatory strategies. A deeper understanding of the dynamic crosstalk between immune-inflammatory processes and BBB integrity is critical for designing targeted interventions that improve neurological outcomes following ischemic stroke.

## 2 Heterogeneity of subtypes in ischemic stroke and immune-BBB injury

Ischemic stroke can be divided into thrombotic, embolic and cerebral venous sinus thrombosis subtypes according to the etiology (7).The pathophysiological differences among these subtypes help elucidate the characteristic patterns of immune-inflammatory activation and BBB disruption (8). This heterogeneity suggests that intervention strategies targeting the ‘immune-BBB axis’ may require personalized adjustments based on specific stroke types (9).

Atherothrombosis is one of the principal subtypes of ischemic stroke, with its pathological core being the rupture of atherosclerotic plaques and subsequent *in-situ* thrombosis (10). The immune-inflammatory response in this subtype exhibits distinctive features, characterized by acute exacerbation superimposed upon a chronic inflammatory baseline (11, 12). Atherosclerotic lesions inherently constitute a chronic, low-grade inflammatory process within the vascular wall (13). When plaques become unstable and rupture, this dormant inflammatory focus becomes rapidly activated, not only triggering acute thrombosis but also intensely mobilizing local innate immune components – particularly macrophages – thereby amplifying the inflammatory cascade (14). Consequently, BBB impairment in this stroke subtype often follows a progressive pattern, closely linked to persistent endothelial dysfunction and plaque instability, which may ultimately complicate the determination of the optimal therapeutic time window (15).

Cardioembolic stroke typically manifests with an abrupt onset, resulting from the sudden occlusion of major cerebral vessels by dislodged emboli (16, 17). This leads to an immediate and complete interruption of cerebral blood flow, precipitating rapid energy failure within the ischemic core and triggering an intense and rapidly evolving inflammatory “storm” (18). A substantial number of peripheral immune cells, particularly neutrophils, are swiftly recruited to the infarcted area, releasing high levels of matrix metalloproteinases and inflammatory cytokines (19). Consequently, BBB disruption occurs early in the process and tends to be severe, which explains why this subtype carries a significantly higher risk of hemorrhagic transformation following revascularization therapy compared to other stroke types (20).

In contrast to arterial ischemic stroke, the core pathology of cerebral venous sinus thrombosis (CVST) centers on impaired venous drainage leading to venous hypertension (21). This fundamental hemodynamic distinction underlies the unique characteristics of its immune response and blood-brain barrier disruption (22). The abrupt rise in venous pressure primarily disrupts Starling forces, triggering distinctive and often more extensive vasogenic edema (23, 24). Immune cell infiltration likely occurs predominantly through the venous end, potentially involving different adhesion molecules and chemokine profiles compared to arterial ischemia (25). Consequently, BBB dysfunction in CVST results from the combined effects of direct mechanical stress from venous hypertension and subsequent inflammatory activation, revealing novel therapeutic targets distinct from those in arterial ischemic stroke (26).

## 3 Mechanisms of immune-inflammatory activation

In ischemic stroke, immune cells such as astrocytes, microglia, oligodendrocytes, neutrophils, T lymphocytes, and monocytes regulate the immune response and maintain BBB function through different mechanisms (27). Specifically, astrocytes and microglia play an important role in the local immune response by secreting cytokines and chemokines (28); oligodendrocytes participate in immune regulation through myelination and neural repair (29); neutrophils, T lymphocytes, and monocytes modulate the immune response and exacerbate BBB damage through various mechanisms (30). With a deeper understanding of the mechanisms underlying these cell types, future therapeutic strategies may focus on targeting the regulation of these cells’ functions to both reduce excessive immune responses and promote neuroprotection and BBB repair (31). Further research will help develop new therapeutic targets for these cell types, providing more effective treatment options.

### 3.1 Central immune cell responses

As essential components of the central nervous system (CNS), microglia, astrocytes, and oligodendrocytes play key roles in post-stroke immune regulation by shaping the microenvironment surrounding the infarct area (32). Microglial polarization, astrocytic end-feet swelling, and the immunomodulation of oligodendrocytes are coordinated events in the CNS immune response. They respectively regulate the inflammatory process, edema formation, and myelin repair. Through complex interaction networks, these cells collectively influence the processes of neuronal injury and recovery (33). Ischemic injury can trigger multi-level repair mechanisms in the CNS, including neurogenesis, synaptic remodeling, vascular reconstruction, and myelin repair, providing new directions for developing therapeutic strategies that promote neurological recovery (34).

#### 3.1.1 Microglia

**Figure 1:**
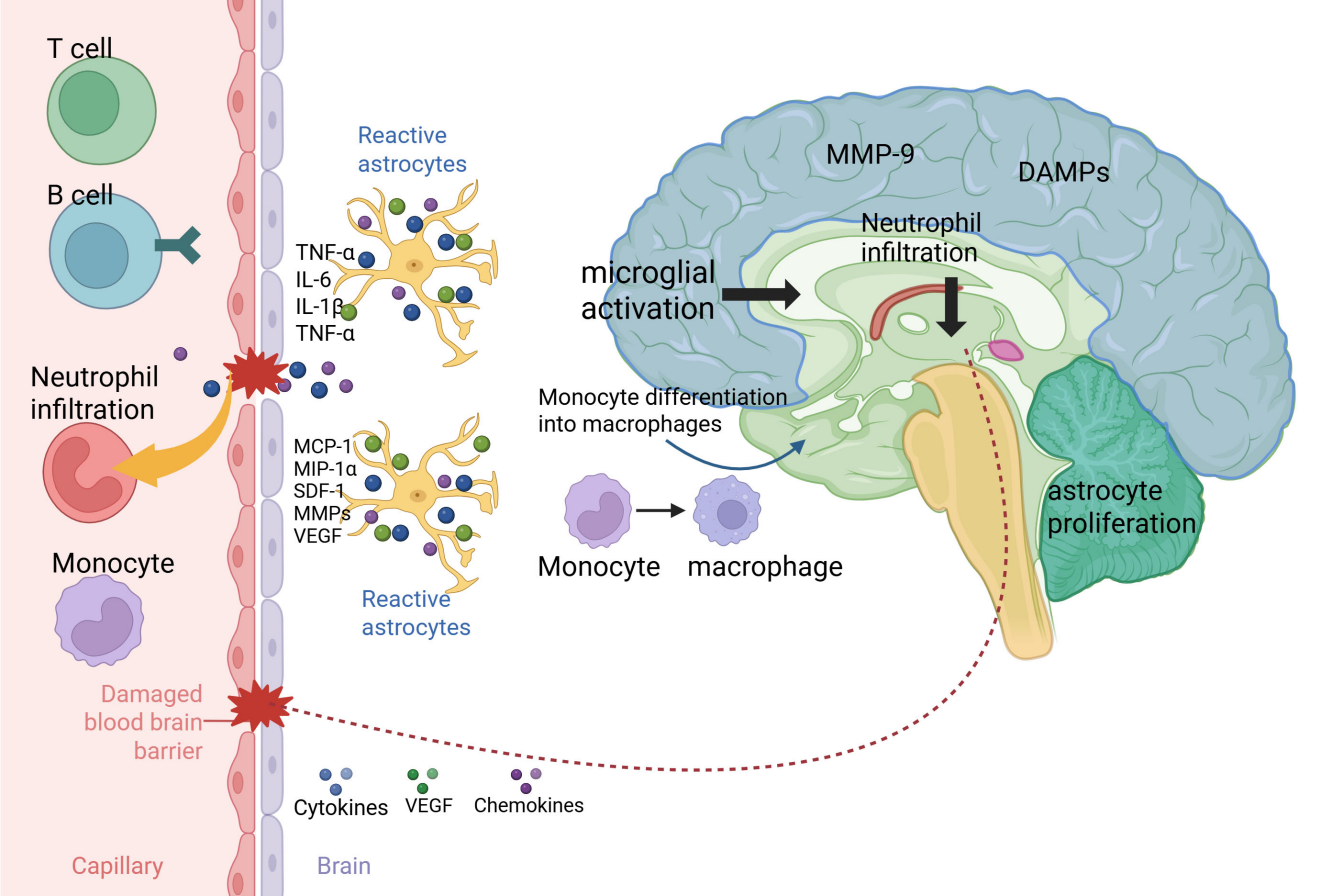
Schematic illustration of immune inflammation and BBB disruption following ischemic stroke. The onset of stroke leads to brain tissue injury and rapidly triggers the activation of glial cells, including the transformation of microglia into an activated state and astrocytes into a reactive state. These activated glial cells release large amounts of pro-inflammatory cytokines (such as TNF-α, IL-6, and IL-1β) and chemokines, creating a highly inflammatory microenvironment locally. This inflammatory cascade results in the breakdown of the blood-brain barrier (BBB), with its structural integrity being disrupted by molecules such as matrix metalloproteinases (MMPs). The compromised BBB acts like an open gate, allowing massive infiltration of peripheral immune cells into the brain. Neutrophils, as the front line of innate immunity, are the first to invade the brain parenchyma. Subsequently, monocytes migrate into the tissue and may further differentiate into macrophages. In the later stages, adaptive immune cells, including T cells and B cells, are also recruited to the lesion area. All of these cellular components engage in complex crosstalk through their secreted molecules—such as cytokines, chemokines, VEGF, and MMPs—forming a self-perpetuating vicious cycle that drives secondary inflammatory injury and ultimately influences disease progression and outcome.

Microglia are the primary immune cells of the CNS, participating in either inflammatory or reparative processes through polarization into distinct phenotypes (31). After ischemic injury, microglia, as the intrinsic immune cells of the central nervous system, can be rapidly activated by ATP released from damaged neurons and surrounding glial cells via the P2X7 receptor pathway, thereby promoting the synthesis and secretion of pro-inflammatory factors (35). In response to the dynamic changes following ischemic injury, microglia are activated into different phenotypes, namely the M1 and M2 phenotypes (36). These two distinct phenotypes also perform different functions: the pro-inflammatory classically activated M1 phenotype exerts neurotoxic effects, while the anti-inflammatory alternatively activated M2 phenotype plays a neuroprotective role (37). The activation of M1-type microglia is regulated by various pro-inflammatory signals. The classical activation pathway is primarily driven by IFN-γ signaling, which binds to IFN-γ receptors on the cell surface and initiates the JAK-STAT1 cascade (38). Under stroke pathological conditions, multiple alternative activation pathways collectively regulate microglial polarization, including the TLR-4/TPAF-6 signaling axis, the TLR-3/IRF-3 pathway, and SYK-dependent signaling mediated by CD8, FcγR, or Clec4 receptors. These diverse signaling pathways ultimately converge on the nuclear translocation of the NF-κB transcription factor, synergistically promoting the upregulation of pro-inflammatory cytokine gene expression (39). M1-type microglia are pro-inflammatory and exacerbate neural damage by releasing inflammatory mediators such as interleukin (IL)-1β, IL-6, tumor necrosis factor-α (TNF-α), and reactive oxygen species (ROS) (**Figure 1**). They play a key role in acute inflammatory responses and neurodegenerative diseases (40). Among them, TNF-α and IL-1β can directly impair neuronal synaptic plasticity. In a mouse model of middle cerebral artery occlusion (MCAO), M1-type microglia significantly exacerbated vascular endothelial injury and blood-brain barrier (BBB) disruption after ischemic stroke by secreting the pro-inflammatory factor TNF-α. Meanwhile, the produced IL-1β directly caused a reduction in synaptic density in the hippocampus and cortical regions, leading to severe cognitive dysfunction (41). Pathologically, M1 polarization predominates the acute inflammatory response and serves as a key effector cell in early neuronal injury following ischemic stroke (42).

As important anti-inflammatory effector cells in the CNS, M2-type microglia are primarily activated under the regulation of Th2-type cytokines (43). At the molecular level, IL-4 and IL-13 bind to their receptors on the cell surface, activating the JAK1/STAT6 signaling pathway, which in turn upregulates the expression of key regulatory molecules such as PPARγ and SOCS, thereby promoting the polarization of microglia toward the M2 phenotype (44, 45). Activated M2-type microglia exhibit significant neurotrophic and anti-inflammatory properties. Their main effector molecules include chitinase-like proteins (Ym1/2), IL-10, IL-4, arginase-1 (Arg-1), and transforming growth factor-β (TGF-β) (41). Anti-inflammatory cytokines such as TGF-β and IL-10 suppress inflammatory responses, while the release of neurotrophic factors like BDNF promotes neuronal survival. Additionally, repair factors such as Arg-1 contribute to tissue remodeling, collectively exerting neuroprotective effects in ischemic stroke (46). The functions of microglia vary at different stages, and some of their roles may even be opposing. In ischemic stroke, M1 and M2 phenotypes can undergo mutual transformation, offering new insights and strategies for therapeutic intervention.

#### 3.1.2 Astrocytes

In ischemic stroke, astrocytes exhibit a dynamic and bidirectional response, initially contributing to BBB dysfunction and edema formation through the release of pro-inflammatory factors and activation of matrix metalloproteinases (MMPs), and later shifting to a neuroprotective phenotype by clearing glutamate, promoting BBB repair, and secreting neurotrophic factors to support tissue recovery (47, 48). Astrocytic end-feet are an essential component of the BBB, and their swelling can lead to vasogenic edema (49). The end-foot structures form tight connections with the cerebral vascular walls via their basal lamina, constituting a critical interface of the neurovascular unit (50).

From a molecular perspective, aquaporin-4 (AQP4) plays a central regulatory role in the process of end-foot swelling (51). AQP4 is polarized distributed on the basal membrane side of astrocytic end-feet, with its expression density significantly higher than in other cellular regions (52). It is precisely this unique distribution that enables AQP4 to precisely regulate the transport of water molecules across the BBB, thereby maintaining water and electrolyte balance in the CNS (53). Under pathological conditions, various factors can lead to dysfunction of AQP4 (51). Following reactive astrogliosis, activated astrocytes synthesize and secrete large amounts of pro-inflammatory cytokines, such as TNF-α and IL-1β (54). These pro-inflammatory cytokines promote AQP4 transcription by activating the NF-κB signaling pathway (55).During the pathological process of ischemic stroke, miR-29 is downregulated, leading to increased AQP4 expression, which exacerbates brain edema and BBB disruption (51). In addition, several other factors also influence AQP4 expression, collectively disrupting normal osmotic balance and contributing to the development of edema.

The formation of cardiovascular-related edema can be divided into three stages. In the first stage, inflammatory cytokines disrupt the structural integrity of the BBB, leading to the extravasation of plasma proteins (56). In the second stage, the extravasated proteins increase the osmotic pressure in the interstitial space, driving the influx of water molecules via AQP4 (57). In the third stage, the accumulation of water molecules leads to mechanical swelling of the astrocytic end-feet, which further compresses microvessels and exacerbates ischemic injury (58). This vicious cycle is observed in various neurological disorders, including traumatic brain injury, ischemic stroke, and hepatic encephalopathy.

From a therapeutic perspective, microRNAs (miRNAs) play a key role in regulating AQP4 expression in astrocytes, offering new targeted strategies for the treatment of brain edema following ischemic stroke (59). miR-130a selectively regulates the expression of the AQP4-M1 isoform, thereby affecting water homeostasis after ischemia (60) The miRNA-AQP4 regulatory network plays a central role in the development of brain edema and provides a theoretical foundation for the development of temporally specific targeted therapeutic strategies.

#### 3.1.3 Oligodendrocytes

Oligodendrocytes are key glial cells in the CNS responsible for the formation and maintenance of myelin sheaths. They are not only essential for efficient nerve signal transmission but also contribute to CNS homeostasis and the regulation of pathological processes through immune modulation, metabolic support, and neuroprotection (61). Due to their high iron content, limited antioxidant capacity, strong reliance on oxidative metabolism, abundant lipid composition, and highly permeable glutamate receptors, oligodendrocytes exhibit unique vulnerability to ischemic injury in the CNS and often undergo irreversible damage (34). Following ischemia, oligodendrocytes undergo glutamate excitotoxicity, leading to intracellular Ca²^+^ overload and mitochondrial dysfunction (62). At the same time, the destruction of the myelin structure leads to the release of antigenic components such as myelin basic protein (MBP), which subsequently activates microglia and infiltrating T cells (63). The expression of MHC class II molecules on the surface of oligodendrocytes is significantly upregulated, enabling them to acquire antigen-presenting capabilities. This further contributes to the immune-inflammatory response in the CNS and exacerbates secondary neuronal damage following ischemia (64). The mechanisms of oligodendrocyte injury following ischemic stroke are complex, involving multiple pathological processes such as excitotoxicity, oxidative stress, and immune inflammation. As a result, therapeutic strategies targeting oligodendrocytes are increasingly diverse and multifaceted (33). In terms of neuroprotection, glutamate receptor antagonists can inhibit Ca²^+^ influx and alleviate excitotoxic damage (65). For immune modulation, MHC class II inhibitors can block the antigen-presenting function of oligodendrocytes (66). Anti-inflammatory cytokines can suppress excessive microglial activation (67); and regulation of the complement system can mitigate complement-mediated myelin damage (68). In promoting myelin repair, LINGO-1 antagonists enhance remyelination by inhibiting the Nogo-A signaling pathway; miRNA regulation can activate myelination programs; and neurotrophic factors support oligodendrocyte survival and enhance their myelination capacity (69).

### 3.2 Peripheral immune cell responses

After ischemic stroke, peripheral immune cells infiltrate the CNS through the compromised blood-brain barrier (BBB), forming a complex immune-inflammatory network (4). These immune cells participate in secondary injury and repair processes after ischemia through various mechanisms, primarily including neutrophils, monocyte–macrophages, and T lymphocytes (70).

#### 3.2.1 Neutrophils

Neutrophils are the first immune cells to arrive at the site of injury after ischemia, rapidly infiltrating brain tissue. They initiate transendothelial migration by binding to intercellular adhesion molecule-1 (ICAM-1) and vascular cell adhesion molecule-1 (VCAM-1) on cerebral endothelial cells through adhesion molecules on their surface (71). During this process, neutrophils release matrix metalloproteinase-9 (MMP-9), which degrades tight junction proteins and components of the basement membrane, further compromising the integrity of the BBB (72). Neutrophils also secrete elastase, increasing vascular permeability and facilitating the extravasation of plasma proteins and inflammatory mediators into the brain parenchyma (73). Infiltrating neutrophils produce reactive oxygen species (ROS) and nitric oxide (NO) via NADPH oxidase, leading to lipid peroxidation and DNA damage in neurons and glial cells (74). Additionally, neutrophils release neutrophil extracellular traps (NETs), which are composed of histones, antimicrobial peptides, and proteases that directly damage surrounding tissues and exacerbate the inflammatory response (75). The formation of NETs depends on peptidylarginine deiminase 4 (PAD4), whose activation promotes the release of citrullinated histones, further amplifying inflammatory signaling (76).

Neutrophils, as the earliest infiltrating immune cells following ischemic stroke, play a critical role in exacerbating neuroinflammation and secondary brain injury through their excessive activation (77). Various therapeutic strategies have been developed to target this pathological process. Neutrophils aggravate microvascular injury after ischemia by obstructing cerebral capillaries. In the acute phase of stroke, targeted clearance or inhibition of neutrophil infiltration—via delivery of anti-Ly6G antibodies or blockade of CXC chemokine receptor 2 (CXCR2) and very late antigen-4 (VLA-4)—can significantly improve neurological deficits and reduce infarct volume (78). In regulating NET formation, PAD4 inhibitors can block histone citrullination, while DNase degrades already-formed NETs. Combined with ROS scavengers such as N-acetylcysteine, these approaches help mitigate NET-mediated tissue damage and inflammation (71, 79). In terms of combating oxidative stress, the use of NADPH oxidase inhibitors and mitochondria-targeted antioxidants can effectively reduce oxidative damage (80).

#### 3.2.2 Monocytes

After ischemic stroke, peripheral blood monocytes are recruited to the injured brain tissue via the CCL2/CCR2 chemokine axis (81). Monocytes can be classified into three subsets based on surface markers: classical (CD14++CD16−), intermediate (CD14++CD16+), and non-classical (CD14+CD16++). Among them, the classical subset exhibits a strong chemotactic response to MCP-1 released from the injury site due to its high expression of CCR2 (82). During their translocation across the BBB, these cells rely on VLA-4 integrin to adhere to the vascular endothelium and secrete MMP-9 to degrade tight junction proteins, thereby further compromising the integrity of the BBB (83). After entering the brain parenchyma, monocytes differentiate into macrophages under the regulation of the local microenvironment, with the pro-inflammatory M1 phenotype activated by signals such as IFN-γ and TNF-α to release inflammatory cytokines and nitric oxide (NO) for antimicrobial functions, while the anti-inflammatory M2 phenotype is induced by IL-4, IL-10, TGF-β, and macrophage colony-stimulating factor (M-CSF) to participate in tissue repair through the production of extracellular matrix components and angiogenic factors (84).

Studies have shown that the proportion of CD14++CD16+ monocytes is significantly associated with patient prognosis, making it a potential biomarker (85). Regarding therapeutic strategies targeting monocytes, inhibition of the CCL2/CCR2 chemokine axis can significantly reduce monocyte infiltration into brain tissue, with enhanced efficacy when combined with VLA-4 antagonists (86). At the level of functional regulation, current strategies include using TREM1 inhibitors to suppress M1 polarization or promoting M2 polarization through IL-4/IL-13-loaded nanoparticles (87).

#### 3.2.3 T lymphocytes

T lymphocytes exhibit complex dynamic changes and dual roles in the pathological process of ischemic stroke (88). Following ischemic stroke, infiltration of CD4^+^ and CD8^+^ T lymphocytes into the injured brain tissue is markedly increased, with a corresponding rise in the proportion of these T cell subsets (89). By day 14 after ischemic stroke, while infiltration of myeloid cells such as neutrophils significantly declines, lymphocyte subsets including CD4^+^ and CD8^+^ T cells persist within the central nervous system, and this sustained presence during the chronic phase may be closely associated with long-term brain injury progression and secondary neurodegeneration (90). These cells contribute to neural injury through multiple mechanisms, with CD8^+^ T cells inducing direct neuronal death via the perforin-granzyme pathway (91). CD4^+^ T cells are generally divided into T helper (Th) cells and regulatory T cells (Treg) (92). CD4^+^ T cells participate in immune regulation after ischemic stroke through differentiation into Th1 and Th2 subsets; Th1 cells promote neuroinflammation by secreting IFN-γ mediated via IP-10, whereas Th2 cells exert anti-inflammatory effects by producing IL-10 and reducing infarct volume, although their precise roles remain controversial in current research (89). Treg cells, without directly infiltrating the brain parenchyma, suppress excessive microglial activation by secreting IL-10, promote oligodendrogenesis by inducing a reparative microglial phenotype through osteopontin secretion, and inhibit pathological astrocyte proliferation via the release of amphiregulin (93).

T cell–targeted therapy has emerged as a critical strategy for immune intervention in ischemic stroke, aiming to modulate T cell function in a multidimensional manner to balance neuroinflammation and tissue repair. Immunosuppressive approaches focus on controlling pathogenic T cell responses, such as the use of Fingolimod to block T cell egress from lymph nodes (94), or the use of anti-VLA-4 antibodies to inhibit T cell infiltration into the brain (95). Immunomodulatory approaches aim to enhance protective functions, such as using low-dose IL-2 to selectively expand Tregs or promoting neurorepair through adoptive transfer of Tregs (96).

### 3.3 Network of inflammatory mediators

The network of inflammatory mediators after ischemic stroke is a complex regulatory system composed of various molecules, including cytokines, chemokines, the complement system, and metabolites (97). These mediators collectively drive the process of secondary neurological injury by activating endothelial cells, recruiting immune cells, and disrupting the BBB.

#### 3.3.1 Cytokines

IL-1β is a key mediator of the inflammatory response following ischemic stroke, primarily produced by activated microglia and infiltrating neutrophils via the NLRP3 inflammasome (98). Its activation is a precisely regulated process: following cerebral ischemia, danger signals such as ATP and mitochondrial DNA released by damaged cells act as initial triggers. These signals activate the NLRP3 inflammasome, which in turn prompts caspase-1 to cleave the inactive pro-IL-1β into the highly biologically active mature IL-1β, which is then released extracellularly (99). Upon release, IL-1β exerts multiple detrimental effects by binding to its receptor IL-1R and transducing signals via the adaptor protein MyD88 (100). By activating downstream signaling pathways, IL-1β reduces the expression of key tight junction proteins, such as occludin and claudin-5, in BBB endothelial cells (101). This disrupts the tight junction structure, thereby increasing BBB permeability and facilitating the infiltration of inflammatory cells and harmful substances. By activating the JNK signaling pathway, IL-1β significantly upregulates the pro-apoptotic protein Bax, subsequently inducing apoptosis in cerebrovascular endothelial cells and ultimately compromising BBB function through dual mechanisms: disrupting structural integrity and promoting cell death (102). Furthermore, IL-1β stimulates cerebral vascular endothelial cells to highly express adhesion molecules such as ICAM-1 and VCAM-1, leading to the substantial recruitment of inflammatory cells like neutrophils to the lesion site, which exacerbates inflammatory infiltration and secondary damage (103).

TNF-α exhibits a time-dependent and biphasic regulatory role in the pathogenesis of ischemic stroke. In the acute phase, it is primarily produced by rapidly activated resident microglia, whereas during the later stages, the sustained expression of TNF-α mainly originates from peripheral macrophages infiltrating the ischemic brain tissue. This process amplifies the neuroinflammatory cascade and exacerbates secondary brain damage (104).This temporal disparity in cellular sources partially influences the functional shift of TNF-α from pro-inflammatory effects to potential protective roles in ischemic stroke. At the molecular level, TNF-α primarily binds to its type I receptor (TNFR1), activating canonical inflammatory signaling pathways such as NF-κB and MAPK. This activation upregulates both the expression and enzymatic activity of MMP-9, leading to degradation of critical structural components in the basement membrane of the blood-brain barrier—particularly type IV collagen—thereby compromising barrier integrity and increasing permeability (105, 106). Moreover, TNF-α disrupts the microstructure of lipid rafts on vascular endothelial cell membranes, interfering with the membrane localization and stable assembly of tight junction proteins such as ZO-1, thereby compromising intercellular sealing integrity. Concurrently, its activation of the RhoA/ROCK signaling pathway induces robust contraction of the endothelial actin-myosin cytoskeleton, resulting in enlarged intercellular gaps and thereby collectively exacerbating vascular hyperpermeability, ultimately leading to severe vascular leakage and cerebral edema (107). TNF-α can also mediate neuroprotective signals via its type II receptor (TNFR2), participating in processes of cell survival and tissue repair (108). This functional duality of mediating both damage and protection underscores its potential as a therapeutic target, the modulation of which must be precisely tailored to the pathological stage and receptor activity.

Beyond IL-1β and TNF-α, numerous other cytokines play significant roles in the pathological progression of ischemic stroke. IL-6 mediates its pro-inflammatory effects in various pathological states, including ischemic stroke, primarily through a mechanism known as “trans-signaling,” which is considered one of its key pathogenic pathways in such inflammatory diseases (109).

Upon formation of the complex between soluble IL-6 receptor (sIL-6R) and IL-6, it binds to the gp130 protein on the surface of endothelial cells (110). This binding subsequently activates the downstream JAK/STAT3 signaling pathway, ultimately leading to increased BBB permeability. Produced mainly by activated γδ T cells and Th17 cells, IL-17 specifically targets the extracellular domain of the tight junction protein Claudin-5 in the BBB, disrupting inter-endothelial connections and serving as a crucial mechanism for BBB damage (111). IFN-γ is primarily secreted by infiltrated cytotoxic T cells and natural killer (NK) cells (112).

Under inflammatory conditions such as cerebral ischemia, it can significantly induce microglia to upregulate the expression of class II MHC molecules (113). This enhances local antigen-presenting capacity, thereby activating specific T-cell immune responses, which promote cytotoxic effects and contribute to delayed inflammatory injury. Together with IL-1β and TNF-α, these inflammatory cytokines form a multi-layered regulatory network that synergistically disrupts the structural integrity of the BBB at multiple points and promotes the progression of post-ischemic brain damage.

#### 3.3.2 Complement system

In ischemic stroke, the complement system is highly activated through both the classical and alternative pathways, contributing synergistically to immune cell recruitment, BBB damage, and subsequent brain injury (114). Activation of the classical complement pathway is initiated by C1q’s recognition of specific molecules on the surface of apoptotic or stressed cells, such as calreticulin, which serves as a canonical “eat-me” signal that facilitates C1q binding (115). This binding subsequently activates C1r and C1s, leading to the cleavage of C4 and C2 and the assembly of the C3 convertase, thereby initiating the classical cascade (116). Meanwhile, the alternative pathway demonstrates a high propensity for spontaneous activation (116). Under ischemic and hypoxic conditions, the expression and function of complement regulatory factors become disrupted. A notable reduction or impairment of endothelial surface proteins CD55 and CD59 leads to diminished control over complement activation, thus promoting an excessive complement response (117). This process drives the C3 molecule into a “ticking over” state, characterized by continuous low-level spontaneous hydrolysis that generates C3b. The generated C3b deposits onto unprotected self-cell walls and, upon binding to factor B, forms the stable C3 convertase (C3bBb) (118). This powerful amplification loop then drastically activates the complement system, leading to massive release of inflammatory factors, formation of membrane attack complexes, and cell lysis, all of which significantly exacerbate brain tissue damage.

The diverse effector fragments generated upon complement activation serve as the core executors of complement-mediated tissue injury. Among them, C3a and C5a are potent anaphylatoxins released during this process. They exert their effects by binding to their specific G-protein-coupled receptors, C3aR and C5aR, respectively (119). C5a can activate pro-inflammatory signaling pathways such as NF-κB and upregulate the expression of adhesion molecules on endothelial cells or macrophages, thereby promoting the adhesion of leukocytes to the endothelium and the recruitment of inflammatory cells (120). C3a and C5a also trigger histamine release from mast cells, resulting in a rapid and direct increase in vascular wall permeability (121). Of paramount importance, C5a—one of the most potent known chemoattractants—exerts a powerful chemotactic effect on neutrophils, resulting in massive inflammatory cell recruitment to the injury site, which in turn initiates a robust inflammatory response (122). The terminal product of the complement cascade, the membrane attack complex (MAC, also known as C5b-9), directly disrupts the integrity of the cell membrane by forming transmembrane pores on target cells such as vascular endothelial cells (123). This action interferes with cellular osmoregulation, promotes a massive influx of key ions like calcium, and ultimately leads to cell swelling, lysis, and death, consequently compromising the structure of the BBB. These effector fragments collectively amplify the secondary pathological processes following ischemic brain injury.

#### 3.3.3 Metabolites

Ischemic stroke leads to a rapid disruption of local metabolic activity, followed by the accumulation of various bioactive intermediate metabolites (124). These metabolites not only reflect an imbalance in energy metabolism but also act as signaling molecules that participate in the dynamic regulation of the brain tissue microenvironment. Lactate exerts complex, phase-dependent effects in ischemic brain injury. During the acute phase, rapidly elevated lactate levels contribute to the inflammatory response post-ischemia (125). Lactate may modulate immune cell function by activating the GPR81 receptor, thereby indirectly influencing the activation process of the NLRP3 inflammasome (126). Lactate, functioning as an endogenous inhibitor of histone deacetylases (HDACs), can regulate the expression of related genes, thereby participating in the modulation of the inflammatory response (127). Acidosis triggered by lactate accumulation can disturb the 3D structure of tight junction proteins, leading to impaired BBB integrity (128). During the subacute phase of the pathology, lactate can be utilized as an alternative energy source by some surviving neurons and astrocytes, while concurrently promoting angiogenesis and tissue repair through receptor-mediated signaling (129). Mitochondrial DNA (mtDNA) is another crucial damage-associated molecular pattern (DAMP). Following ischemic-induced abnormal opening of the mitochondrial permeability transition pore (mPTP), mtDNA is released into the cytoplasm, where it can be recognized by the pattern recognition receptor TLR9, thereby activating the production of type I interferons and the STING signaling pathway, and driving a distinct interferon-associated neuroinflammatory response (130). High concentrations of extracellular ATP, a critical purinergic signal released during tissue injury, activate the P2X7 receptor, thereby promoting NLRP3 inflammasome activation and compromising endothelial cell integrity, further aggravating neuroinflammation (131). In contrast, adenosine, the degradation product of ATP, typically exerts anti-inflammatory effects via the A2a receptor. However, at excessively high concentrations, it paradoxically exacerbates vascular leakage, thereby illustrating its concentration-dependent effects (132). Ketone bodies (e.g., β-hydroxybutyrate) and short-chain fatty acids (e.g., butyrate) primarily exhibit protective functions. Specifically, β-hydroxybutyrate can effectively inhibit the activation of the NLRP3 inflammasome, and preclinical studies have demonstrated its ability to reduce BBB permeability (133). Butyrate, by inhibiting histone deacetylases (HDAC), upregulates the expression of tight junction proteins such as occludin, thereby reinforcing the BBB (134). The balance of these metabolites collectively determines the ultimate fate of the ischemic brain tissue.

### 3.4 Distinctions between innate and adaptive immune responses

Following ischemic stroke, brain tissue is initially affected by disrupted energy metabolism and oxidative stress, which subsequently activate both central and peripheral immune systems (135). However, different types of immune responses exhibit significant differences in timing, mechanisms, and their effects on the BBB (4). Grasping these differences is essential for designing precise interventions and determining the optimal timing for therapy.

Innate immune responses are the earliest defense mechanisms triggered after ischemia, typically initiating within minutes to hours (136). Key cellular participants include microglia, astrocytes, neutrophils, and macrophages (137). In the ischemic environment, microglia tend to polarize toward the M1 phenotype, releasing pro-inflammatory factors such as IL-1β and TNF-α, while also activating oxidative stress and matrix metalloproteinases, leading to degradation of tight junction proteins and endothelial dysfunction (15, 138). Neutrophils rapidly migrate to the infarcted area, traversing the endothelium via adhesion molecules (ICAM-1, VCAM-1) and releasing reactive oxygen species and proteases, further exacerbating vasogenic edema (139, 140). At this stage, the primary role of the innate immune system is the clearance of necrotic cells and pathogens, but it inevitably causes acute BBB disruption (30).

Adaptive immune responses, in contrast, gradually dominate during the subacute phase, typically becoming prominent 1–3 days after ischemia and potentially persisting for several weeks (92). Key cells include CD4^+^ and CD8^+^ T cells as well as B cells, with regulatory T cells (Tregs) playing a crucial role in limiting inflammation and promoting repair (141, 142). Adaptive immunity primarily modulates the inflammatory response through specific cytokines (such as IFN-γ and IL-17) and antibody-mediated mechanisms (143). Compared to innate immunity, adaptive immune responses have a weaker direct destructive effect on the BBB, but by sustaining or prolonging the inflammatory environment, they may contribute to secondary BBB injury while also providing signals for BBB repair and neural remodeling (144).

In summary, post-stroke immune responses display clear temporal and functional division: innate immunity reacts rapidly and directly mediates BBB damage, whereas adaptive immunity intervenes later, maintaining inflammation or facilitating repair (27). Recognizing this dynamic distinction is critical for selecting appropriate intervention strategies at different time points, such as inhibiting MMP-9 and pro-inflammatory cytokines during the acute phase, and modulating T cell activity in the subacute phase to support BBB restoration (145).

## 4 Pathological process of BBB disruption

**Figure 2:**
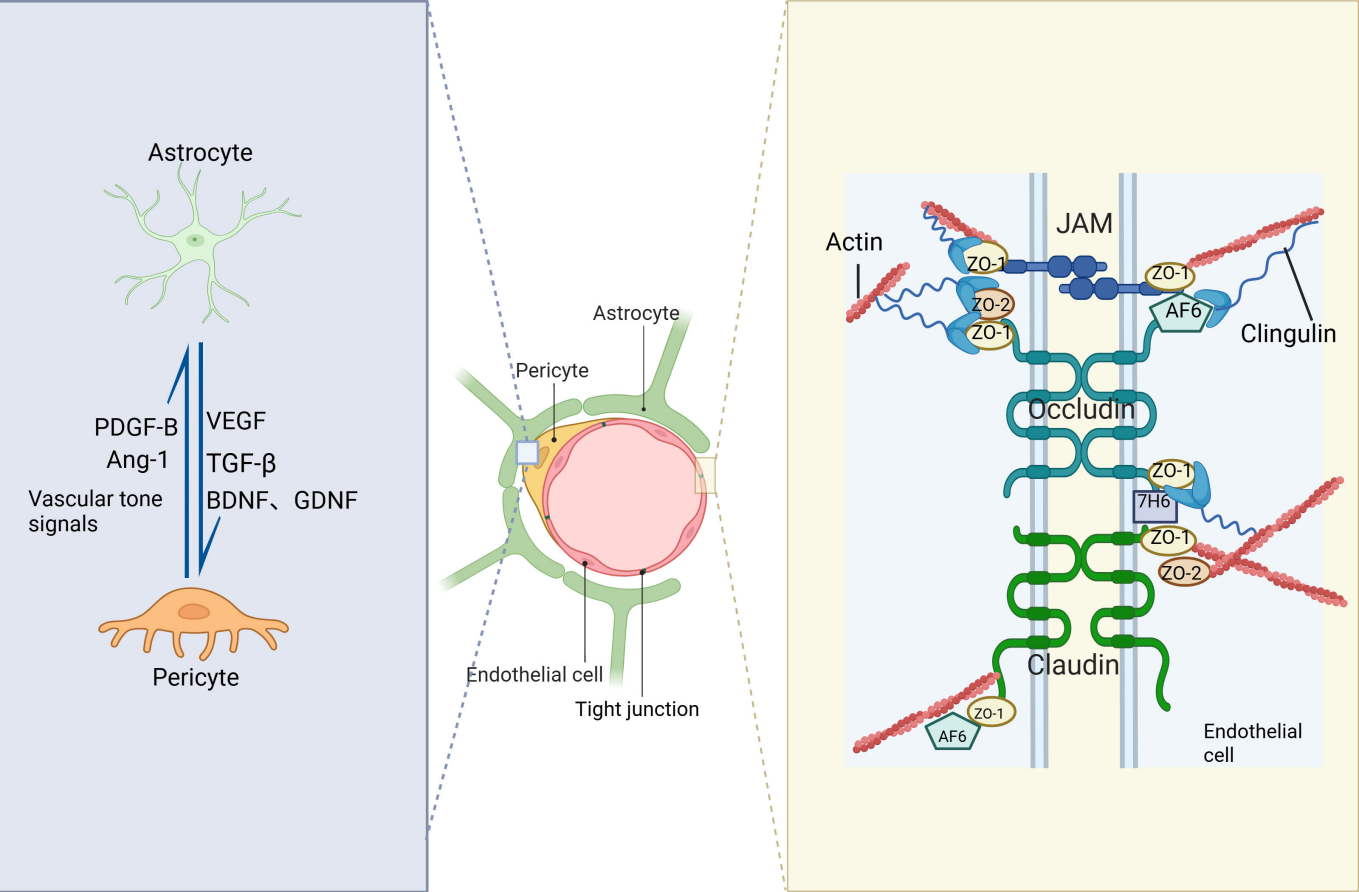
The structural and molecular composition of the BBB. At its core lies the precise cooperation within the neurovascular unit, composed of endothelial cells, pericytes, and astrocytic endfeet. Astrocytes and pericytes interact by secreting various substances to maintain the stability of the blood-brain barrier. Astrocytes primarily secrete VEGF, TGF-β, and BDNF to regulate vascular permeability and support barrier function, while pericytes secrete PDGF-B, Ang-1, and extracellular matrix components to strengthen the vascular structure and enhance the barrier, with these signals reciprocally influencing each other to ensure proper blood-brain barrier function. Tight junctions between cerebral capillary endothelial cells serve as the fundamental structural basis for the barrier function. These junctions are formed by transmembrane proteins such as Occludin and Claudin, which act like “seams” sealing the intercellular space, and are firmly anchored to the actin cytoskeleton via intracellular scaffold proteins, thereby strictly restricting paracellular transport. Pericytes, embedded within the basement membrane and enveloping the endothelial cells, participate in the regulation of blood flow and barrier maintenance. Meanwhile, astrocytic endfeet extensively cover the abluminal surface of blood vessels, providing nutritional support and assisting in permeability regulation. This multilayered cellular and molecular architecture collectively establishes a highly selective semipermeable barrier that permits the passage of nutrients while effectively preventing toxins, pathogens, and inflammatory cells from entering brain tissue, thereby ensuring the homeostasis of the central nervous system microenvironment.

The BBB is not a passive or static barrier, but rather a highly dynamic and functionally complex physiological interface system (146). It is constituted by brain microvascular endothelial cells and their tight junctions, the basement membrane enveloping the endothelial cells, pericytes, and astrocytic endfeet, collectively forming a highly specialized structure that precisely regulates the exchange of substances between the central nervous system and peripheral circulation, thereby maintaining the absolute homeostasis of the cerebral microenvironment (**Figure 2**). Under various pathological conditions, such as ischemic stroke, traumatic brain injury, neurodegenerative diseases, and central nervous system infections, the integrity of the BBB is compromised, triggering a cascade of detrimental events. The pathological processes involved are profound and complex, and can be systematically characterized by three major aspects: structural disruption, functional dysregulation, and temporospatial dynamics with significant clinical implications.

### 4.1 Structural disruption

The structure of the BBB underpins its function. Any damage to its physical integrity constitutes the initiating event of pathological processes, with tight junctions and the basement membrane being the two primary targets of attack. Tight junctions between brain microvascular endothelial cells represent the principal structural component restricting paracellular permeability; they form a complex protein network composed of transmembrane proteins and cytoplasmic scaffolding proteins (147). Accessory proteins such as zonula occludens (ZO)-1, ZO-2, AF-6, 7H6, and cingulin function as essential molecular bridges connecting transmembrane tight junction proteins to the intracellular actin cytoskeleton, thereby playing a critical role in preserving the structural and functional integrity of tight junctions (148).

Under pathological conditions, two major effector molecules—MMP-9 and ROS—are markedly activated and produced in large quantities (149). Ischemia, inflammation, and other pathological stimuli lead to intracellular calcium overload, which activates a cascade of signaling pathways that promote the conversion of pro-MMP-9 into its active proteolytic form (148). MMP-9 can specifically recognize and hydrolyze defined peptide bonds within tight junction proteins such as ZO-1 and Occludin, leading to their structural disruption and functional loss (150). Meanwhile, energy metabolism disorders and reperfusion injury result in excessive production of ROS (151). ROS not only directly oxidize tight junction proteins, inducing conformational changes and functional impairment, but also upregulate the expression and activity of MMP-9, thereby establishing a positive feedback loop that markedly accelerates the disintegration of tight junctions (152). Once key junctional proteins are disrupted, gaps form between endothelial cells, resulting in the loss of BBB selective permeability and effectively opening the gateway for the uncontrolled leakage of macromolecules (146). Beneath the tight junctions, the basement membrane provides essential structural support and polarization signals to endothelial cells. It is primarily composed of type IV collagen, laminin, fibronectin, nidogen, and basement membrane proteoglycans, forming a dense and intricate meshwork (153). MMP-9 also exhibits potent degradative activity against basement membrane components, with laminin and fibronectin serving as its primary substrates (154). The loss of laminin weakens endothelial cell adhesion and survival signaling, while the degradation of fibronectin directly compromises the mechanical integrity of the basement membrane (155). The basement membrane becomes sparse, discontinuous, and even exhibits defects, rendering the vascular structure fragile and more prone to collapse or aneurysmal dilation. Furthermore, the disrupted basement membrane serves as a direct migratory pathway for inflammatory cell infiltration, thereby exacerbating the inflammatory response within the brain parenchyma (156). Therefore, damage to the basement membrane represents a critical step in the progression and irreversibility of blood-brain barrier structural disruption.

### 4.2 Functional dysregulation

The structural integrity of the blood-brain barrier (BBB) forms the basis of its function, which is far from being a passive defense; rather, it constitutes an active and precise regulatory system. Consequently, structural collapse inevitably leads to comprehensive functional dysregulation, and the failure of these regulatory mechanisms profoundly disrupts the homeostasis of the brain tissue microenvironment (157). BBB endothelial cells express a variety of specific transporters that actively regulate the uptake of essential nutrients and the clearance of metabolic waste, thereby maintaining cerebral homeostasis. Injury-related signals, such as inflammatory cytokines TNF-α and IL-1β, can significantly downregulate the expression and function of glucose transporter 1 (GLUT-1) (138). he brain relies almost entirely on glucose for energy production; therefore, downregulation of GLUT-1 directly compromises energy supply to neural cells, exacerbating metabolic stress and cell death in both neurons and glial cells, thereby contributing to a vicious cycle of injury (158). In addition, the function of P-glycoprotein (P-gp), which is responsible for the efflux of xenobiotics, toxins, and drugs from the brain back into the bloodstream, is also severely impaired (159). Although P-gp may be transiently upregulated in the acute phase, sustained injury ultimately suppresses its expression and function, leading to the failure of P-gp–mediated efflux and the accumulation of neurotoxic compounds in the brain (160).

Vasogenic edema is the most lethal and urgent consequence of BBB functional dysregulation and represents a critical factor in the clinical deterioration of patients (161). Under normal conditions, large plasma proteins are strictly confined within the vascular lumen, maintaining colloid osmotic pressure balance across the vascular wall. When tight junctions and the basement membrane are disrupted, vascular permeability dramatically increases, leading to the non-selective leakage of protein-rich plasma components into the extracellular space of the brain (162). The influx of macromolecules such as albumin significantly increases the colloid osmotic pressure of the interstitial fluid in brain tissue, drawing large amounts of water from the intravascular space into the parenchyma and resulting in vasogenic cerebral edema (163). Cerebral edema leads to a rapid increase in intracranial pressure (ICP), which in turn compresses normal brain tissue and cerebral blood vessels, resulting in reduced cerebral perfusion pressure (CPP) and secondary ischemic injury (164). In severe cases, elevated ICP can cause brain herniation, posing an immediate threat to life. Therefore, controlling vasogenic edema is one of the primary therapeutic goals during the acute clinical phase.

### 4.3 Temporal dynamics

The temporal dynamics of BBB disruption are critically important for determining treatment timing and developing intervention strategies, which has led to the classification of ischemic stroke into acute and subacute phases. In the acute phase, BBB disruption is primarily triggered by the initial insult, with energy depletion causing ion pump dysfunction, leading to cytotoxic edema and the activation of multiple enzymatic pathways (165). Excitotoxicity causes massive calcium influx, activating calcium-dependent proteases and NOS, which rapidly trigger MMP-9 activation, leading to the swift degradation of tight junctions and the basement membrane (166). Therefore, the leakage observed during the acute phase reflects the direct effects of the initial injury, is typically severe, and closely correlates with the earliest neurological deficits and peak edema formation (167).

During the subacute phase, a complex cellular inflammatory response is initiated. The leaked plasma components themselves act as potent inflammatory stimuli, recruiting neutrophils and monocytes from the peripheral circulation to infiltrate the brain parenchyma through the compromised BBB (168). These activated immune cells release a new and more extensive wave of pro-inflammatory cytokines (e.g., TNF-α, IL-1β, IL-6), chemokines, and proteases—including MMP-9, MMP-3, and MMP-12 (73). This secondary inflammatory response, mediated by immune activation rather than the initial ischemic insult, launches a second wave of BBB disruption characterized by a renewed surge in permeability, which not only impairs barrier self-repair but also expands the final infarct size and is pathologically linked to secondary neuronal apoptosis and long-term neurological deficits (169).

The biphasic nature of BBB disruption reveals the existence of multiple therapeutic time windows. Acute-phase interventions should focus on controlling the mechanisms of primary injury, including thrombolysis, neuroprotection, and early inhibition of matrix metalloproteinases. In contrast, subacute-phase strategies should prioritize anti-inflammatory and immunomodulatory approaches to effectively halt secondary injury processes and promote neural tissue repair.

## 5 Role of gut microbial metabolites in ischemic brain injury

In recent years, the cross-system interaction known as the “gut–brain axis” has emerged as a critical modulator in the pathophysiology of ischemic stroke. Cerebral ischemia not only triggers local neuroinflammation and neuronal damage, but also, via autonomic nervous imbalance, reduced intestinal perfusion, and elevated systemic inflammatory mediators, compromises gut barrier function and disrupts the composition of the gut microbiota (170, 171). Alterations in both the abundance and functionality of intestinal microbes lead to dramatic fluctuations in the levels and spectra of gut-derived metabolites (172). These metabolites, in turn, can feedback into the brain through the bloodstream, immune system signaling, or vagal nerve pathways, thereby influencing central immune responses and the homeostasis of the BBB (173). Consequently, microbial metabolite–mediated regulation via the gut–brain axis represents an important extension of the ischemia–immune–BBB injury cascade, making it a cutting-edge direction in the field of neuroimmunology (174).

### 5.1 Disruption of intestinal homeostasis and microbiota alterations after stroke

Acute cerebral ischemia can rapidly induce dysregulation of intestinal neural control, including reduced gut motility, decreased blood perfusion, and enhanced epithelial stress (175). These changes lead to decreased expression of tight-junction proteins in the intestinal mucosa, resulting in increased barrier permeability (176). Barrier disruption, on the one hand, facilitates the entry of bacteria-associated molecules (such as LPS) into the bloodstream, and on the other hand, causes shifts in the composition of the microbiota: dominant short-chain fatty acid (SCFA)–producing bacteria decline, while opportunistic pathogens increase in proportion (177, 178). Animal studies have demonstrated that short-chain fatty acid (SCFA) levels decrease during the early phase after stroke, and stroke-induced gut dysbiosis is associated with metabolic alterations linked to enhanced inflammatory signaling (179, 180). This altered metabolic profile can persist for days to weeks, suggesting that gut dysbiosis may span the entire disease course and influence immune status in distant organs (181).

### 5.2 Regulation of immune responses by microbial metabolites

Among the numerous metabolites produced by gut microbiota, short-chain fatty acids (SCFAs) and tryptophan derivatives have attracted the most research attention (182). SCFAs can bind to G protein-coupled receptors (e.g., GPR41, GPR43) to modulate inflammatory responses in both peripheral and central systems (183). They suppress the release of pro-inflammatory factors from macrophages and microglia, reduce NF-κB signaling activity, and promote regulatory T cell generation via HDAC inhibition, thereby alleviating secondary inflammatory damage in the brain (184–186). In animal models of stroke, exogenous supplementation of SCFAs has been shown to partially reverse excessive immune activation, suggesting their potential to modulate the inflammatory microenvironment (179).

Tryptophan metabolism displays a complex bidirectional role in post-stroke immune regulation (187). Gut bacteria-derived indole metabolites can activate the aryl hydrocarbon receptor (AhR), which helps attenuate astrocyte inflammation and modulate microglial activity (188). Following stroke, however, the kynurenine pathway tends to become dysregulated, leading to the accumulation of neurotoxic metabolites such as 3-hydroxykynurenine (3-HK) and quinolinic acid (189). These compounds can exacerbate immune responses and induce oxidative stress, thereby contributing to additional tissue damage (190). Collectively, these alterations in tryptophan metabolism represent a key regulatory node in post-ischemic immune pathology (191, 192).

### 5.3 Effects of microbial metabolites on blood–brain barrier structure and function

Metabolites derived from gut microbiota not only regulate inflammatory responses but also directly affect the reconstruction and stability of the BBB (193). Studies have shown that short-chain fatty acids (SCFAs) can enhance the expression of tight junction proteins in brain microvascular endothelial cells, thereby reducing barrier permeability (194). The underlying mechanisms may involve improvements in mitochondrial energy metabolism, suppression of ROS production, and stabilization of the cytoskeleton, ultimately strengthening endothelial resistance to hypoxic stress (195).

Conversely, certain pro-inflammatory metabolites can exacerbate BBB disruption. For instance, lipopolysaccharide (LPS) can upregulate MMP-9 via TLR4 signaling, leading to degradation of tight junction proteins and potentially triggering endothelial pyroptosis, further impairing barrier function (196, 197). Metabolites such as trimethylamine N-oxide (TMAO) increase VCAM-1 and ICAM-1 expression, facilitating adhesion and migration of neutrophils and monocytes, thus amplifying neuroinflammation (198, 199). Neurotoxic compounds generated through the kynurenine pathway can also compromise the integrity of the neurovascular unit, widening the extent of leakage (200). Collectively, these findings indicate that microbial metabolic alterations influence the BBB at multiple levels, thereby impacting the severity of ischemic brain injury and the potential for recovery (170).

### 5.4 The theoretical significance of gut-derived signals in the progression of brain injury

Integrating gut microbial metabolites into the pathological framework of ischemic brain injury provides a renewed understanding of how peripheral conditions influence central nervous system responses (171). Traditional models primarily emphasize local inflammation and endothelial impairment, whereas the metabolic signaling axis between the gut and the brain helps explain why individuals with comparable degrees of cerebral injury may exhibit markedly different immune reactions and patterns of blood–brain barrier disruption (201). From a research perspective, short-chain fatty acids, tryptophan metabolites, and their relative ratios may serve as promising biomarkers (202). From an interventional standpoint, modulating the gut microbiota or its metabolic pathways could represent a potential adjunctive therapeutic approach.

Overall, gut-derived microbial metabolites exert cross-system, long-distance regulatory effects, participating in immune activation, barrier homeostasis, and the propagation of neuroinflammation during ischemic brain injury (203). As these mechanisms become clearer, stroke may increasingly be viewed not as an isolated cerebral event, but as a complex pathological process shaped by the body’s interconnected systemic networks (204).

## 6 Therapeutic strategies

Given the complex pathophysiology and temporal dynamics of BBB disruption, effective treatment requires a phase-specific, multi-targeted approach. Therapeutic strategies should aim not only to limit acute-phase injury and stabilize BBB integrity but also to modulate immune responses and support long-term repair mechanisms during the subacute and chronic stages.

### 6.1 Anti-inflammatory targeted interventions

Following ischemic stroke, cerebral ischemia and hypoxia trigger a robust inflammatory response involving the activation of various cytokines, inflammasomes, and chemokines. These inflammatory mediators not only exacerbate neuronal injury but also contribute to BBB disruption, leading to cerebral edema and hemorrhagic transformation. Therefore, anti-inflammatory therapies targeting these pathways have emerged as key strategies for mitigating secondary brain injury. Anti-adhesion therapy primarily utilizes monoclonal antibodies to inhibit β2-integrin function, thereby interfering with the adhesion and migration processes between leukocytes and endothelial cells (205). Cytokine-targeted therapy focuses on IL- 1β, employing neutralizing antibodies or receptor antagonists to block its signaling pathway, which helps alleviate inflammatory responses and preserve blood-brain barrier integrity (206). Additionally, matrix metalloproteinase inhibitors are administered in the later acute phase to broadly suppress MMP-9 activity, mitigating degradation of the basement membrane and tight junction proteins (207). Statins act on HMG-CoA reductase and, when administered at high doses during the acute phase, not only exert lipid-lowering effects but also demonstrate anti-inflammatory, antioxidant, and endothelial protective properties, contributing to the stabilization of the blood-brain barrier (208). To address oxidative stress, free radical scavengers neutralize ROS, thereby reducing cellular damage (209). In terms of tissue repair, activation of the Mas receptor promotes the Angiotensin-(1-7) pathway, counteracting the adverse effects induced by Ang II and exhibiting anti-inflammatory and anti-fibrotic activities (210). In cell-based therapy, mesenchymal stem cells release a variety of bioactive factors through paracrine mechanisms, coordinating immune regulation, neuroprotection, and vascular remodeling, thereby modulating the functional balance of the immune-inflammatory–blood-brain barrier axis (211). Meanwhile, microglial modulators target signaling pathways such as TREM2, driving the polarization of microglia from the pro-inflammatory M1 phenotype toward the anti-inflammatory and reparative M2 phenotype, thus attenuating neural tissue damage (212). **Table 1** provides a summary of pharmacological agents targeting the immune inflammation–BBB axis.

**Table 1:** Summary of therapies targeting the immune inflammation–BBB axis after ischemic stroke.

Category	Target	Role	Therapeutic strategy	References
Anti-adhesion Therapy	β2-Integrin	Mediating the adhesion and migration of leukocytes with endothelial cells.	Using monoclonal antibodies to block its function(anti β2-Integrin).	(205)
Cytokine Targeted Therapy	IL-1β	Key pro-inflammatory factors that drive the inflammatory response and disrupt the blood-brain barrier.	Using neutralizing antibodies or receptor antagonists to block the IL-1β signaling pathway.	(213)
MMP Inhibitors	MMP-9	Degrading basement membrane and tight junction proteins.	Using broad-spectrum inhibitors to suppress its activity in the later stages of the acute phase.	(150)
Statins	HMG-CoA Reductase	It has multiple effects, including lipid-lowering, anti-inflammatory, antioxidant, eNOS upregulation, and endothelial protection.	High-dose statins are used in the acute phase to exert their pleiotropic effects and stabilize the blood-brain barrier.	(214)
Free Radical Scavengers	ROS	Causing oxidative stress, damaging endothelial cells, neurons, and tight junctions	Using oxygen free radical scavengers (e.g., SOD, glutathione) reduces oxidative stress, protecting endothelial cells, neurons, and tight junctions from damage.	(209)
Prorepair Mediators	Mas receptor [for Ang-(1-7)]	Counteracts the damaging effects of Ang II, with anti-inflammatory, anti-fibrotic, and reparative properties.	Angiotensin-(1-7) activates Mas receptor–mediated protective pathways in endothelial cells, neurons, and glial cells, counteracting Ang II–induced inflammation, oxidative stress, and tissue damage.	(215)
Cell Therapy	Multiple	Mesenchymal stem cells coordinate anti-inflammatory, neuroprotective, angiogenic, and reparative processes by secreting various factors.	Mesenchymal stem cells regulate the immune-inflammatory-BBB axis through paracrine effects.	(216)
Microglia Modulators	TREM2/Inflammatory Pathways	Excessive activation of microglia produces pro-inflammatory factors, exacerbating damage.	Drugs (e.g., IL-4, resveratrol) can shift overactivated microglia from pro-inflammatory M1 to anti-inflammatory, reparative M2, reducing inflammation and tissue damage.	(217)

#### 6.1.1 Cytokine inhibitors

Cytokines play a central role in the inflammatory response following ischemic stroke, with interleukin-1 (IL-1) serving as a key pro-inflammatory mediator that activates astrocytes and microglia, promotes inflammatory cell infiltration, and facilitates neuronal apoptosis (218). Interleukin-1 receptor antagonists (e.g., Anakinra) competitively bind to IL-1 receptors, blocking downstream signaling pathways and thereby inhibiting the inflammatory response (219). Animal studies have shown that Anakinra significantly reduces infarct volume and improves neurological deficits in the middle cerebral artery occlusion (MCAO) model in rats (220). In the Anakinra-treated group, brain tissue levels of IL-1β were reduced, neutrophil infiltration was attenuated, and BBB integrity was preserved (219). Moreover, Anakinra has demonstrated a relatively broad therapeutic window, with significant neuroprotective effects observed even when administered up to 12 hours after ischemic onset (219). These preclinical studies provide a solid foundation for the clinical application of Anakinra, and several early-phase clinical trials are currently evaluating its safety and efficacy in stroke patients.

#### 6.1.2 NLRP3 inflammasome inhibitors

The NLRP3 inflammasome is a multiprotein complex that becomes activated following ischemic stroke, promoting the maturation and release of IL-1β and IL-18, thereby exacerbating neuroinflammation (221). MCC950, a potent inhibitor of the NLRP3 inflammasome, significantly reduces levels of IL-1β and IL-18 in brain tissue and attenuates neutrophil infiltration in mouse models of ischemic stroke (222). MCC950 maintains BBB integrity and alleviates BBB damage by inhibiting MMP-9 expression and preserving tight junction proteins (223). MCC950 also reduces cerebral edema and the risk of hemorrhagic transformation, thereby improving long-term neurological recovery (224). These findings suggest that targeting the NLRP3 inflammasome may represent a promising therapeutic strategy, particularly for acute-phase intervention in ischemic stroke.

#### 6.1.3 Chemokine blockade

Chemokines play a critical role in regulating immune cell migration and infiltration, with CCL2 (monocyte chemoattractant protein-1, MCP-1) serving as a key chemokine that recruits monocytes and macrophages to ischemic brain tissue, thereby exacerbating inflammatory responses and BBB disruption (225). CCL2-neutralizing antibodies reduce monocyte infiltration and activation by blocking CCL2 activity. In animal studies, treatment with CCL2 antibodies significantly decreased the number of inflammatory cells in the ischemic brain region, alleviated cerebral edema, and reduced neuronal damage (226). Although anti-CCL2 antibodies have shown promising effects in preclinical studies, their clinical application still requires further optimization to minimize the risk of systemic immunosuppression.

### 6.2 BBB protection and repair

#### 6.2.1 Tight junction stabilizers

Tight junctions are a crucial component of the BBB, and their dysfunction leads to increased vascular permeability. ZO-1, a key tight junction protein, has been closely associated with BBB disruption due to its downregulated expression (227). The Rho-associated coiled-coil containing kinase (ROCK) signaling pathway is activated following ischemic stroke and can suppress ZO-1 expression, thereby disrupting tight junction integrity (227). Fasudil dichloroacetate (FDCA) is a ROCK inhibitor that maintains blood-brain barrier integrity by suppressing MMP-9 expression and preventing the degradation of ZO-1 and occludin, thereby alleviating microglia-mediated neuroinflammation (228). Preclinical data support the therapeutic potential of FDCA in ischemic stroke, and small-scale clinical trials have confirmed its safety and partial efficacy, providing a foundation for subsequent large-scale applications (229).

#### 6.2.2 Vascular protective agents

Vascular stability is essential for BBB function. The Ang-1/Tie2 signaling pathway is a key regulator in maintaining vascular integrity and stability (230). Ang-1 enhances endothelial cell survival, reduces vascular permeability, and suppresses inflammatory responses by activating the Tie2 receptor. In ischemic stroke models, agonists of the Ang-1/Tie2 signaling pathway have demonstrated protective effects by attenuating BBB damage, reducing cerebral edema, and promoting angiogenesis and vascular repair (231). Furthermore, activation of Tie2 signaling can inhibit NF-κB–mediated inflammatory responses, thereby reducing cytokine release and immune cell infiltration (232). The combined use of Ang-1/Tie2 agonists with other anti-inflammatory agents, such as IL-1 inhibitors, may produce synergistic effects and further enhance therapeutic outcomes.

## 7 Challenges and prospects

Despite the promising therapeutic potential of targeting neuroinflammation and preserving BBB integrity in ischemic stroke, several formidable challenges hinder clinical translation. At the same time, these obstacles highlight innovative directions for future interdisciplinary research (233). One of the primary challenges lies in the precise temporal modulation of immunotherapies (234). Neuroinflammation is a highly dynamic and phase-dependent process (235). During the acute phase of ischemic stroke, excessive inflammatory responses contribute to secondary brain injury and BBB disruption, necessitating early and effective anti-inflammatory interventions (236). However, in the subacute and recovery phases, specific immune components—such as alternatively activated M2 microglia and regulatory cytokines—play beneficial roles in debris clearance, neurotrophic support, and vascular regeneration (237). Therefore, uniform or non-specific anti-inflammatory strategies may produce contradictory effects depending on the timing of administration (238). Future therapies must be intelligently tailored to the temporal dynamics of post-stroke inflammation (239).

A second major challenge is interindividual heterogeneity, which underscores the need for personalized treatment strategies (240). Patient stratification based on reliable biomarkers is essential for identifying those most likely to benefit from specific interventions (241). For instance, peripheral blood levels of MMP-9—a key protease involved in BBB degradation—can serve as a dynamic indicator of BBB injury severity (242). This biomarker may guide therapeutic decisions regarding the use of MMP inhibitors or potent anti-inflammatory agents, thereby avoiding ineffective or inappropriate treatments in unselected patient populations (243).

Looking forward, technological innovation and multidisciplinary integration will be pivotal in overcoming these translational bottlenecks. On the experimental front, the development of stem cell-derived human brain microvascular organoids and “BBB-on-a-chip” systems offers physiologically relevant *in vitro* models that recapitulate the cellular complexity and functional properties of the human BBB (244). When co-cultured with immune cells, these platforms enable mechanistic studies of neurovascular-immune interactions and facilitate high-throughput drug screening (245). Concurrently, artificial intelligence (AI) presents transformative opportunities in predictive medicine (246). Machine learning algorithms can integrate multi-omics datasets, neuroimaging results, and pharmacological databases to predict patient-specific drug responses and optimize dosing regimens (247). These approaches may significantly accelerate the pace of drug development and enhance clinical decision-making (248). In summary, by combining temporal precision, biomarker-guided personalization, and technological advances such as organoid modeling and AI-based analytics, we may systematically address the current limitations in stroke therapeutics. This integrated strategy holds promise for developing more effective and precise treatments for patients suffering from ischemic stroke.
